# Utilizing solar energy to improve the oxygen evolution reaction kinetics in zinc–air battery

**DOI:** 10.1038/s41467-019-12627-2

**Published:** 2019-10-18

**Authors:** Xiaorui Liu, Yifei Yuan, Jie Liu, Bin Liu, Xu Chen, Jia Ding, Xiaopeng Han, Yida Deng, Cheng Zhong, Wenbin Hu

**Affiliations:** 1 Key Laboratory of Advanced Ceramics and Machining Technology (Ministry of Education), School of Materials Science and Engineering, Tianjin University, 300072 Tianjin, China; 2 Joint School of National University of Singapore and Tianjin University, International Campus of Tianjin University, 350207 Binhai New City, Fuzhou China; 3 Department of Mechanical and Industrial Engineering, University of Illinois at Chicago, Chicago, IL 60607 USA; 4 State Key Laboratory of Metal Matrix Composites, Department of Materials Science and Engineering, Shanghai Jiao Tong University, 200240 Shanghai, China

**Keywords:** Batteries, Batteries

## Abstract

Directly harvesting solar energy for battery charging represents an ultimate solution toward low-cost, green, efficient and sustainable electrochemical energy storage. Here, we design a sunlight promotion strategy into rechargeable zinc–air battery with significantly reduced charging potential below the theoretical cell voltage of zinc–air batteries. The sunlight-promoted zinc–air battery using BiVO_4_ or α-Fe_2_O_3_ air photoelectrode achieves a record-low charge potential of ~1.20 and ~1.43 V, respectively, under illumination, which is lowered by ~0.5–0.8 V compared to the typical charge voltage of ~2 V in conventional zinc–air battery. The band structure and photoelectrochemical stability of photoelectrodes are found to be key factors determining the charging performance of sunlight-promoted zinc–air batteries. The introduction of photoelectrode as an air electrode opens a facile way for developing integrated single-unit zinc–air batteries that can efficiently use solar energy to overcome the high charging overpotential of conventional zinc–air batteries.

## Abstract (web summary)

The authors here report a sunlight-promoted rechargeable zinc–air battery in which photoelectrode is used as the air electrode to substantially lower the charge potential under illumination. Notably, the battery can be initially charged with an extremely low voltage of ~1.20 V.

## Introduction

Solar cell and rechargeable metal–air battery are two totally different systems converting energy of different forms into electricity. Intrinsically, solar cell can only convert light into instant electricity for immediate usage but cannot store energy^1^. Rechargeable metal–air battery, on the other hand, can store (and release) energy repeatedly as needed via oxygen evolution reaction (OER) but this process is essentially plagued by the large overpotential caused by sluggish OER kinetics^2^. Therefore, a combination of these two battery systems would theoretically guarantee a clean and energy-efficient functional integrity, which is however, poorly explored so far.

Among the very few reported studies^1,3–7^, lithium–oxygen battery has been the mostly focused system to be successfully loaded with a photo-responsive electrode where the in situ generated holes possessing positive charges directly^5^ or indirectly^4^ catalyze the OER. Therefore, the notorious large overpotential of the OER has been efficiently suppressed via this synergistic process where solar energy is nominally stored into the rechargeable battery system and further consumed to compensate the energy needed for battery charge. Yet, the high cost and poor safety issues of lithium–oxygen battery make it unsuitable to be coupled with the widely commercialized solar cells aiming at low cost and reliable safety, which thus drives the search for appropriate battery systems.

Among the candidacy of various rechargeable battery technologies, rechargeable zinc–air batteries have attracted great interest thanks to their high energy density (1086 Wh kg^−1^ in theory), environmental friendliness, earth-abundance of Zn, low cost, and excellent safety enabled by the use of aqueous electrolytes^8,9^. Although primary zinc–air batteries have been commercialized^10,11^, the development of rechargeable zinc–air batteries is still in the early stages with several major challenges that should be addressed. One of the most significant challenges is the high charge overpotential of the battery, which is caused by the practical charge voltage typically above 2 V despite the calculated theoretical voltage of 1.65 V^8^. Therefore, the energy efficiency is generally lowered below 60%^9,12^. Besides, the high charge voltage results in the possible degradation of air electrode due to the side reactions during the charging process, greatly reducing the cycle life of rechargeable zinc–air batteries^13,14^. The large charge overpotential is essentially caused by the sluggish kinetics of oxygen evolution reaction (OER) process that proceeds through four proton-coupled electron transfer steps at the air electrode during the charging process^15^. Therefore, the improvement of the OER kinetics is the central scheme for the further development and commercialization of rechargeable zinc–air battery. In this sense, it should be beneficial to effectively harvest the solar energy using the zinc–air battery system and meanwhile to reduce the charge potential with input electric energy.

In present work, we report a sunlight-promoted rechargeable zinc–air battery by integrating semiconductor photoelectrode as air electrode to significantly lower the charge potential by ~0.5–0.8 V under sunlight illumination. By utilizing two typical semiconductor photoelectrodes (i.e., BiVO_4_ and α-Fe_2_O_3_) with different band structures, the effect of the band structure of the semiconductor photoelectrode on the charging performance of the zinc–air battery has been clearly demonstrated and the corresponding sunlight-promoted charging mechanism has been proposed. BiVO_4_- and α-Fe_2_O_3_-based sunlight-promoted zinc–air batteries exhibit an ultra-low initial charge potential of 1.20 and 1.43 V, respectively, even lower than the theoretical cell voltage of zinc–air batteries. Sunlight-promoted zinc–air batteries with α-Fe_2_O_3_ photoelectrode show robust cycling stability due to the high photoelectrochemical stability of α-Fe_2_O_3_ electrode. The concept of sunlight-promoted OER in rechargeable zinc–air battery offers an effective strategy to store the in situ converted energy from the solar cell system and more importantly, to enhance the OER kinetics of zinc–air battery toward its wide application in future.

## Results

### Working mechanism of sunlight-promoted rechargeable zinc–air battery

**Fig. 1:**
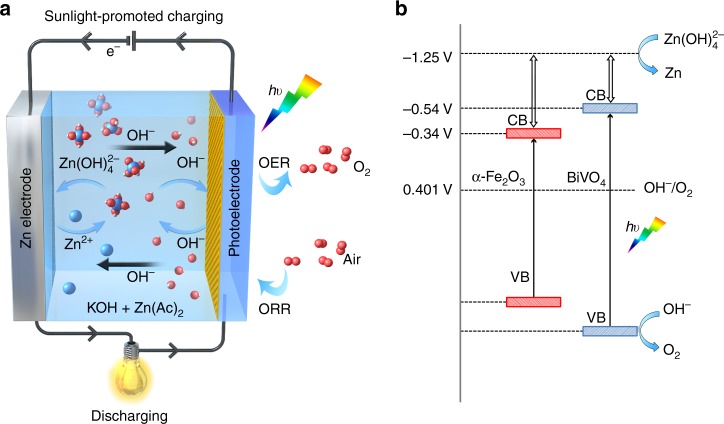
Schematic sunlight-promoted charge and discharge processes of the sunlight-promoted zinc–air battery. **a** The scheme of the basic structure and working principle of the sunlight-promoted rechargeable zinc–air battery. **b** The proposed mechanism of the sunlight-promoted charging process under solar light illumination

Figure 1a schematically illustrates the basic structure and working mechanism of the sunlight-promoted zinc–air battery. It consists of a Zn electrode and a semiconductor photoelectrode (i.e., BiVO_4_ or α-Fe_2_O_3_) as an air electrode assembled in alkaline electrolyte (Fig. 1a). The discharging process resemble that in a conventional zinc–air battery: electrochemical oxidation of Zn to Zn^2+^ on the Zn electrode accompanied by the reduction of oxygen on the air electrode gives electricity output^9,16^. The charging process is associated with OER on the air electrode, which has intrinsically sluggish reaction kinetics, resulting in the large overpotential and low energy efficiency. The relevant mechanism of OER is considered as the following processes:^17^ OER reaction begins with the adsorption of OH^–^ and then form adsorbed OH species (reaction 1). The following step is the reaction of OH^–^ with the adsorbed OH species to produce H_2_O and adsorbed atomic O and the release of an electron (reaction 2). After that, the step in the sequence involves the reaction of an OH^–^ with an adsorbed O atom to form adsorbed OOH species (reaction 3), which then undergo reaction with additional OH^–^ (reaction 4), leading to the formation of adsorbed O_2_ and H_2_O and the release of an electron. Adsorbed O_2_ then desorbs in the last step of the sequence (reaction 5).

1 $${\mathrm{M}} + {\mathrm{OH}}^ - \to {\mathrm{M - OH}} + {\mathrm{e}}^ -$$

2 $${\mathrm{M - OH}} + {\mathrm{OH}}^ - \to {\mathrm{M - O}} + {\mathrm{e}}^ - + {\mathrm{H}}_2{\mathrm{O}}$$

3 $${\mathrm{M - O}} + {\mathrm{OH}}^ - \to {\mathrm{M - OOH}} + {\mathrm{e}}^ -$$

4 $${\mathrm{M - OOH}} + {\mathrm{OH}}^ - \to {\mathrm{M - O}}_2 + {\mathrm{e}}^ - + {\mathrm{H}}_2{\mathrm{O}}$$

5 $${\mathrm{M - O}}_2 \to {\mathrm{M}} + {\mathrm{O}}_2$$

In comparison with OER process in conventional zinc–air battery, the charging process of sunlight-promoted rechargeable zinc–air battery under illumination is different and significantly facilitated due to the formation of photogenerated holes that have strong oxidative ability and are favorable for proton removal. During the charging process under solar light illumination, the photoelectrode absorbs photons from the light source and generates electron–hole pairs. Then the photogenerated electrons are rapidly injected into the conduction band (CB) of semiconducting photoelectrode and further transferred to the Zn electrode through the external circuit, resulting in the reduction of ${\mathrm{Zn}}\left( {{\mathrm{OH}}} \right)_4^{2{\mathrm{ - }}}$ to Zn and OH^−^. The photoexcited holes simultaneously migrate to the photoelectrode surface to oxidize water to oxygen. The reactions for a sunlight-promoted zinc–air battery during discharging and charging are illustrated in Eqs. (6)–(12):

Discharge process:^18,19^

6 $${\mathrm{Zinc}}\,{\mathrm{electrode}}: {\,\,}{\mathrm{Zn}} + 4{\mathrm{OH}}^{\mathrm{ - }} \to {\mathrm{Zn}}\left( {{\mathrm{OH}}} \right)_4^{2{\mathrm{ - }}} \,+ \, 2{\mathrm{e}}^ - E^0 \\ = {\mathrm{ - }}1.25\,{\mathrm{V}}\,{\mathrm{versus}}\,{\mathrm{SHE}}$$

7 $${\mathrm{Zn}}\left( {{\mathrm{OH}}} \right)_4^{2 - } \,\to {\mathrm{ZnO}} + {\mathrm{H}}_2{\mathrm{O}} + 2{\mathrm{OH}}^ -$$

8 $${\mathrm{Air}}\,{\mathrm{electrode}}:{\,} {\mathrm{O}}_2 + 2{\mathrm{H}}_2{\mathrm{O}} + 4{\mathrm{e}}^ - \to 4{\mathrm{OH}}^ - E^0 \\ = 0.401\,{\mathrm{V}}\,{\mathrm{versus}}\,{\mathrm{SHE}}$$

9 $${\mathrm{Overall}}:2{\mathrm{Zn}} + {\mathrm{O}}_2 \to 2{\mathrm{ZnO}}\,E^0 = 1.65\,{\mathrm{V}}$$

Charge process:

10 $${\mathrm{Zinc}}\,{\mathrm{electrode}}:{\mathrm{Zn}}\left( {{\mathrm{OH}}} \right)_4^{2 - } +\, 2{\mathrm{e}}^ - \to {\mathrm{Zn}} + {\mathrm{OH}}^ -$$

11 $${\mathrm{Air}}\,{\mathrm{electrode}}:{\mathrm{Photoelectrode}}\mathop{\longrightarrow}\limits^{{hv}}{\mathrm{e}}^ - + {\mathrm{h}}^ +$$

12 $$4{\mathrm{OH}}^ - + 4{\mathrm{h}}^ + \to 2{\mathrm{H}}_2{\mathrm{O}} + {\mathrm{O}}_2$$

This mechanism is expected to reduce the charge potential of a zinc–air battery through the photooxidation reaction by photogenerated holes to facilitate the OER reaction in the sunlight-promoted charging process. The requirement of photoelectrode in the sunlight-promoted rechargeable zinc–air battery for conversion of OH^−^ to O_2_ should fulfill the essential condition that the valence band (VB) potential lies higher than the O_2_/OH^−^ couple potential (0.401 V vs. standard hydrogen electrode (SHE)). As illustrated in the proposed sunlight-promoted charging mechanism (Fig. 1b), the photovoltage generates when the photoelectrode undergoes photoexcitation, which compensates the high charge potential of the zinc–air battery. It is estimated that theoretical sunlight-promoted charge potential equals the potential difference between the ${\mathrm{Zn}}\left( {{\mathrm{OH}}} \right)_4^{2{\mathrm{ - }}}$/Zn redox potential and the quasi-Fermi level (*E*_F_) of electrons, which, at its most negative situation, approaches to the conduction band minimum (CBM), in the semiconductor photoelectrode. Based on the above discussion, the band structure (e.g., band edge position and band gap) of the photoelectrode significantly affects the charging performance of the sunlight-promoted zinc–air battery. To better understand the mechanism of sunlight-promoted charging process, for the first time, two typical semiconductor photoelectrodes with different band structures (i.e., BiVO_4_ and α-Fe_2_O_3_) were investigated simultaneously as air electrodes for zinc–air batteries.

### Characterization of the photoelectrodes for sunlight-promoted zinc–air battery

**Fig. 2:**
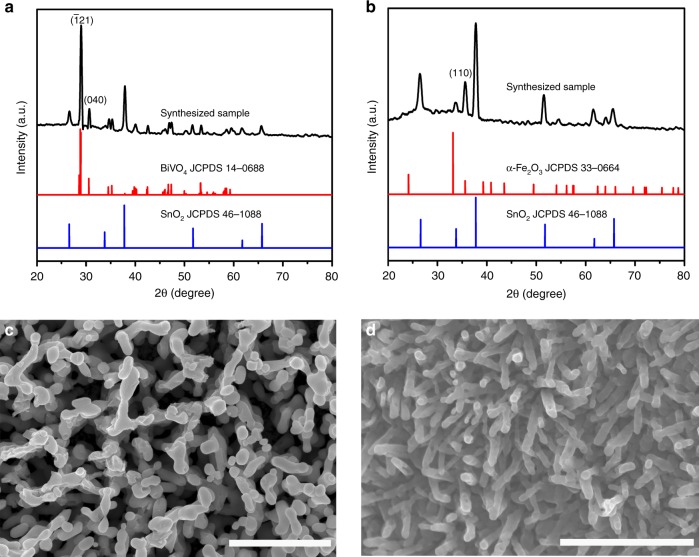
Characterization of the photoelectrodes. XRD patterns of **a** BiVO_4_ film and **b** α-Fe_2_O_3_ film. SEM images of **c** BiVO_4_ and **d** α-Fe_2_O_3_. Scale bars in **c**, **d** are 1 μm

The crystal structures of BiVO_4_ and Fe_2_O_3_ photoelectrodes are analyzed in Fig. 2a, b, respectively. The BiVO_4_ photoelectrode exhibits a structure identical to monoclinic scheelite BiVO_4_ (JCPDS Card No. 14–0688)^20^. The Fe_2_O_3_ photoelectrode is indexed to α-phase Fe_2_O_3_ (JCPDS Card No. 33–0664)^21^. Strong (110) diffraction peak implies preferential growth of hematite nanorods in the [110] direction^22^. All the other peaks are indexed to be SnO_2_ (JCPDS Card No. 46–1088) from the fluorine-doped tin oxide (FTO) substrate. The surface chemical compositions of both BiVO_4_ and α-Fe_2_O_3_ are confirmed by X-ray photoelectron spectroscopy (XPS). Apparently, the characteristic spin–orbit split of the Bi 4f_5/2_ and Bi 4f_7/2_ signals (Supplementary Fig. 1a) and V 2p_1/2_ and V 2p_3/2_ signals ((Supplementary Fig. 1b) were observed, and they correspond to the typical monoclinic scheelite BiVO_4_^23^. In addition, in the survey scan of XPS shown in Supplementary Fig. 1d, e, Fe and O signals can be clearly resolved to represent α-Fe_2_O_3_^24^. Scanning electron microscopy (SEM) reveals that the obtained BiVO_4_ product consists of interconnected columnar-like particles with an average particle size of ~110 nm, forming a three-dimensional (3D) porous network (Fig. 2c). The thickness of the BiVO_4_ film is estimated to be ~650 nm according to the cross-sectional SEM (Supplementary Fig. 2a). SEM image in Fig. 2d shows that the α-Fe_2_O_3_ film grown on the FTO substrate is composed of nanorods with an average diameter of ~70 nm. Cross-sectional SEM image indicates that α-Fe_2_O_3_ nanorods are aligned roughly vertically to the substrates and yield an average length of ~350 nm (Supplementary Fig. 2b).

**Fig. 3:**
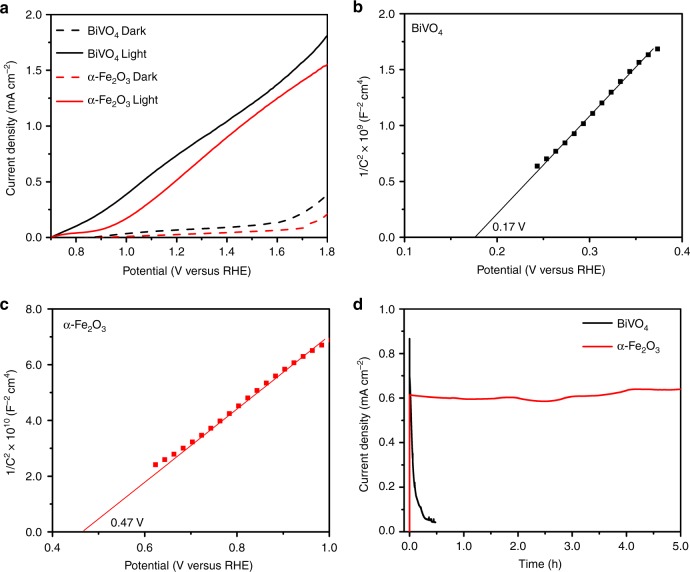
Photoelectrochemical performance of the photoelectrodes. **a** Current density–potential curves of BiVO_4_ and α-Fe_2_O_3_ measured in 1 M KOH (pH = 13.6) in the dark and under AM 1.5 G, 100 mW cm^–2^ illumination. Mott–Schottky plots of the **b** BiVO4 and **c** α-Fe_2_O_3_ at an AC frequency of 1 kHz in the dark. **d** Current density–time curves of the BiVO_4_ and α-Fe_2_O_3_ photoelectrodes under illumination at 1.23 V vs. RHE

Figure 3a shows the current density–potential curves for the OER on BiVO_4_ and α-Fe_2_O_3_ electrodes in the dark and under AM 1.5 G simulated solar light at 100 mW cm^−2^. Both BiVO_4_ and α-Fe_2_O_3_ electrodes exhibit small current density and high onset potential (defined as the potential where photocurrent reaches 0.1 mA cm^−2^) above ~1.4 V for water oxidation in the dark condition. In sharp contrast, the BiVO_4_ and α-Fe_2_O_3_ electrodes show very low onset potential below ~0.9 V under simulated sunlight illumination. Furthermore, the photocurrent increases linearly with the increasing voltage and achieves high values of 0.79 and 0.60 mA cm^−2^ at 1.23 V vs. RHE, respectively, which are more than one order of magnitude larger than those under the dark condition. This indicates that the application of photoelectrodes dramatically accelerate water oxidation kinetics under illumination. The charge-transfer properties of the photoanodes were further investigated by electrochemical impedance spectroscopy (EIS) (Supplementary Fig. 3). In dark, both photoanodes show one semicircle reflecting the charge-transfer resistance at the electrode surface (Supplementary Fig. 3a)^25,26^. Under illumination, Nyquist plots show two semicircles for both photoanodes. The arcs observed in the high and the low frequencies correspond to the bulk and surface charge-transfer processes, respectively^26,27^. The semicircles obtained under illumination have much smaller radius than those in the dark, indicating much smaller charge-transfer resistance due to increased carrier density when the semiconductor photoelectrodes absorbs photons, confirming the photo-responsive property of the photoelectrodes. Moreover, BiVO_4_ electrode has a smaller semicircle radius than α-Fe_2_O_3_ electrode, implying its better charge-transfer efficiency.

To evaluate the band structure of BiVO_4_, Mott–Schottky (M–S) analysis was performed at 1 kHz (Fig. 3b, c). The positive slope of the M–S plots suggests n-type semiconducting nature of both BiVO_4_ and α-Fe_2_O_3_ electrodes. The flat band potential (*E*_FB_) and donor densities (*N*_D_) of two electrodes are calculated based on the slopes of M–S plots^28^, which are 0.17 V (vs. RHE) and 2.18 × 10^20^ cm^−3^ for BiVO_4_ and 0.47 and 1.25 × 10^19^ cm^−3^ for α-Fe_2_O_3_. It should be noted that the M–S analysis is derived from a planar electrode model^29^ and therefore these values are only for the purpose of relative comparison. Compared to α-Fe_2_O_3_ the negative shift of *E*_FB_ and larger *N*_D_ of BiVO_4_ electrode indicate that the Fermi level is closer to the CB^30,31^, leading to more significant band bending in the space charge region than that of α-Fe_2_O_3_ due to a larger difference between the Fermi level of BiVO_4_ photoelectrode and the redox potential of the electrolyte. This facilitates the charge separation efficiency of the photogenerated charge carriers^32,33^, which is consistent with the higher photocurrent density of BiVO_4_ electrode compared to α-Fe_2_O_3_ electrode (Fig. 3a).

The photoelectrochemical stability is another important factor affecting the life of the batteries for practical applications, which was investigated by chronoamperometry measurements under illumination as shown in Fig. 3d. Clearly, α-Fe_2_O_3_ photoelectrode exhibits excellent stability in alkaline electrolyte, retaining nearly 100% of its original activity after 5 h. In addition, the photocurrent density–potential curve of the α-Fe_2_O_3_ photoelectrode remains almost unchanged after the stability test (Supplementary Fig. 4b), confirming its high stability during OER. However, the BiVO_4_ photoelectrode suffers 84.6% decrease in the photocurrent density within only 10 min of operation, indicating the severe photocorrosion in the BiVO_4_ photoelectrode. The structure and morphology of two samples after the stability test are further explored. There is no noticeable change in XRD pattern of α-Fe_2_O_3_ after stability test (Supplementary Fig. 5b). The overall morphology of α-Fe_2_O_3_ is well maintained (Fig. 2d vs. Supplementary Fig. 5d). On the contrary, the XRD peak intensity of the BiVO_4_ photoelectrode remarkably decreases after the stability test (Supplementary Fig. 5a). The SEM image after stability test also shows the dissolution of BiVO_4_ (Supplementary Fig. 5c), suggesting the existing electrode photocorrosion. This suggests that the quick decrease in the photocurrent of BiVO_4_ compared to α-Fe_2_O_3_ under illumination is attributed to the photocorrosion of BiVO_4_ photoelectrode.

### Band structure of the photoelectrode

**Fig. 4:**
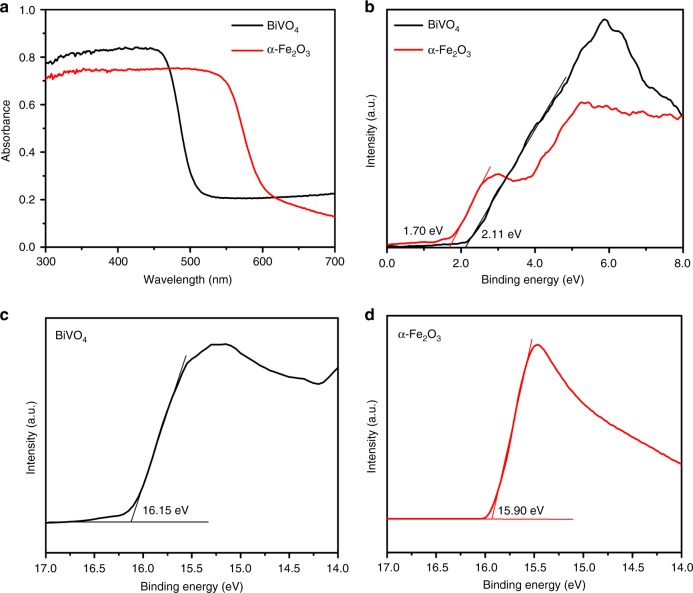
Band structures of the photoelectrodes. **a** Ultraviolet–visible absorption spectra of the BiVO_4_ and α-Fe_2_O_3_ photoelectrodes. **b** XPS valence band spectra of the BiVO_4_ and α-Fe_2_O_3_ photoelectrodes. Work function data of **c** BiVO_4_ and **d** α-Fe_2_O_3_ are from UPS measurements

The optical absorption properties of BiVO_4_ and α-Fe_2_O_3_ photoelectrodes were investigated using ultraviolet–visible (UV–vis) absorption spectra as shown in Fig. 4a. Both photoelectrodes show obvious absorption of visible light. The absorption edge of BiVO_4_ and α-Fe_2_O_3_ extends to 504 nm and 590 nm, corresponding to the band gap energy (*E*_g_) of 2.46 and 2.10 eV, respectively (Supplementary Fig. 6). The VB positions of the two photoelectrodes were measured by X-ray photoelectron spectroscopy (XPS) (Fig. 4b). The positions of the valence band maximum (VBM) relative to the Fermi level (*E*_F_) can be estimated by linear extrapolation of the leading edges in the spectra^34^, which are deduced to be 2.11 and 1.70 eV for BiVO_4_ and α-Fe_2_O_3_, respectively.

To further analyze the band structure, the surface work function of α-Fe_2_O_3_ and BiVO_4_ was measured using ultraviolet photoelectron spectroscopy (UPS). The work function of BiVO_4_ and α-Fe_2_O_3_ is 5.05 and 5.30 eV, respectively (Fig. 4c, d). Furthermore, the UPS data is combined with the values of *E*_g_ and VBM to calculate the positions of the CB edge^35^, which lies below 0.26 V and 0.46 V reference to SHE for BiVO_4_ and α-Fe_2_O_3_, respectively (assuming SHE to be positioned 4.44 eV below the vacuum energy). Based on the charging mechanism mentioned above, the CB edge combined with ${\mathrm{Zn}}\left( {{\mathrm{OH}}} \right)_4^{2{\mathrm{ - }}}$/Zn redox potential can be used to predict the theoretical charge voltage of the sunlight-promoted zinc–air batteries, as presented in Fig. 1b. This will be further discussed and confirmed in combination with performance studies of sunlight-promoted zinc–air batteries.

### The performance of sunlight-promoted zinc–air battery

**Fig. 5:**
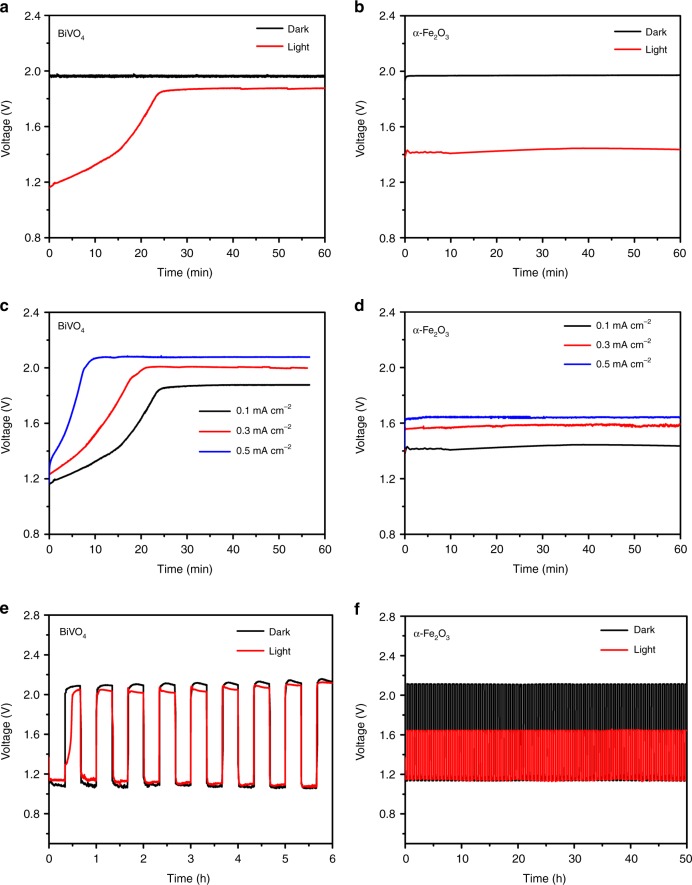
Electrochemical characterizations of the sunlight-promoted rechargeable zinc–air battery. The charging curves of a zinc–air battery in the dark and under illumination with **a** BiVO_4_ and **b** α-Fe_2_O_3_ air photoelectrode, respectively, at a current density of 0.1 mA cm^−2^. The charging curves of a Zinc–air battery with **c** BiVO_4_ and **d** α-Fe_2_O_3_ air photoelectrode, respectively, at the current density of 0.1, 0.3, and 0.5 mA cm^−2^ under illumination. Cycling performance of sunlight-promoted rechargeable zinc–air battery in the dark and under illumination with **e** BiVO_4_ and **f** α-Fe_2_O_3_ air photoelectrode, respectively, at a current density of 0.5 mA cm^−2^

To demonstrate the potential application of photoelectrode as an air electrode, we assembled a single-body-structured zinc–air battery using the semiconductor photoelectrode. The charging curves of zinc–air battery in the dark and under illumination based on BiVO_4_ and α-Fe_2_O_3_ photoelectrodes are shown in Fig. 5a, b. As expected, the introduction of the light dramatically reduces the charge potential of the zinc–air battery. Upon illumination, the charge voltage of BiVO_4_-based zinc–air battery achieves a very low initial charging voltage of ~1.20 V corresponding to the voltage reduction of ~0.76 V (Fig. 5a), which translates to energy savings of close to 38.8% (Supplementary Table 1). This charging voltage value is significantly lower than the theoretical working voltage (1.65 V) of the zinc–air battery, and is also a record-low value among the reported literature to the best of our knowledge. However, the charge potential keeps increasing to get close to the charge potential without illumination, which is attributed to the photocorrosion of BiVO_4_ electrode (Fig. 3d). For α-Fe_2_O_3_-based zinc–air battery under illumination, the charge potential is also obviously lower than the theoretical working potential of the zinc–air battery with a remarkable reduction of ~0.54 V (Fig. 5b), corresponding to energy savings of 27.4% (Supplementary Table 1). Due to the high photoelectrochemical stability of α-Fe_2_O_3_ electrode, the battery exhibits a stable charging plateau under illumination. Moreover, the charging voltage of the battery increases instantly once the light is off while the charging potential drops rapidly with the illumination (Supplementary Fig. 7), confirming the achievement of such a low charge potential owing to the effective utilization of the solar energy and also suggesting the battery possesses a fast light-response. This significant decrease in the charge potential can be related to the band structure of the BiVO_4_ and α-Fe_2_O_3_ (Fig. 1b). Both the negative shift of the onset oxidation potential (Fig. 3a) and the lowering of the charge potential are attributed to the negative shift of the quasi-Fermi level of photoelectrodes compared to the OH^–^/O_2_ redox potential under illumination. Since the OER process is achieved by the photogenerated holes in photoelectrode rather than direct oxidation of OH^–^ under illumination, BiVO_4_ with a more negative CBM exhibits lower charge potential at the initial charging stage. Ideally, the band edge position is pH-dependent and follows the Nernstian pH dependence in aqueous solutions (a change of the pH value shifts the potential by ~59 mV per pH^36,37^). Based on the estimated CBM value (–0.54 V for BiVO_4_; –0.34 V for α-Fe_2_O_3_ at electrolyte pH = 13.6) and the discussion in Fig. 1b, the theoretical charge potential of the sunlight-promoted zinc–air battery based on BiVO_4_ and α-Fe_2_O_3_ is expected to be 0.71 and 0.91 V, respectively. The initial charge potential of BiVO_4_-based zinc–air battery is ~0.23 V lower than the α-Fe_2_O_3_-based one, which matches well with the theoretical value of 0.20 V. Considering the factors including energy gap between the photoelectrode CBM and the *E*_F_ of electrons under working condition, the charge recombination occurring in the photoelectrode–electrolyte interface, and the internal series resistance within the device, it is reasonable to obtain a higher actual charging voltage than the theoretical value.

Figure 5c, d show the sunlight-promoted charging curves at different current densities, where the polarization degree does not drastically increase. Even with the current density increased by 5 times to 0.5 mA cm^−2^, the initial charging voltage (~1.35 V) of BiVO_4_-based battery is still much lower than the theoretical working voltage (1.65 V) of the zinc–air system; α-Fe_2_O_3_-based battery gives a larger charging potential of ~1.64 V, which is very close to the theoretical working voltage. The discharge–charge polarization curves for zinc–air battery using BiVO_4_ and α-Fe_2_O_3_ as the air electrode with and without light illumination are shown in Supplementary Fig. 8. The light illumination significantly decreases the charge potential for both batteries especially at low current densities, resulting from the remarkably improved OER performance under illumination (Fig. 3a). Besides, BiVO_4_ delivers a lower charging voltage compared to α-Fe_2_O_3_ due to the higher OER photoelectrocatalytic activity of BiVO_4_. BiVO_4_- and α-Fe_2_O_3_-based batteries exhibit similar discharge behaviors in dark and under illumination, which is consistent with the oxygen reduction reaction (ORR) performance of the two photoelectrodes (Supplementary Fig. 9). To demonstrate the practical applications of the zinc–air batteries, the batteries were tested via long-term galvanostatic discharge (Supplementary Fig. 10). The initial discharge voltage platforms of the two batteries are similar, in accordance with the similar ORR performance between BiVO_4_ and α-Fe_2_O_3_ electrodes (Supplementary Fig. 9). BiVO_4_-based battery shows distinct decrease in the voltage after discharge of ~10 h while α-Fe_2_O_3_-based battery is relatively stable during the whole discharge for 30 h. This is attributed to the different chemical stability of BiVO_4_ and α-Fe_2_O_3_ in the electrolyte, suggesting the applicable features of α-Fe_2_O_3_-based zinc–air battery. Based on the consumed zinc electrode, the discharge gravimetric capacity and energy density of zinc–air battery using the α-Fe_2_O_3_ are 598.7 mAh g_Zn_^−1^ and 694.5 mWh g_Zn_^−1^ at 0.5 mA cm^−2^ with 46 mg consumed Zn. The gravimetric capacity and energy density of BiVO_4_-based zinc–air battery are 538.5 mAh g_Zn_^–1^ and 540.1 mWh g_Zn_^−1^ respectively, at 0.5 mA cm^–2^ with 39 mg consumed Zn. To investigate the cycling stability, the discharge–charge tests were performed in the dark and under illumination separately. As shown in Fig. 5e, upon illumination, zinc–air battery based on the BiVO_4_ air photoelectrode exhibits a low charge voltage of ~1.30 V at the first 3 min, corresponding to an extremely small discharge–charge voltage gap of ~0.16 V and a very high energy efficiency of ~87.7%. Unfortunately, the charging voltage quickly increases to ~2.12 V and the discharging voltage also gradually decreases to ~1.07 V after 6 h test, corresponding to the increase of voltage gap to 1.05 V and the decrease of energy efficiency to 50.5% after the test. The observed performance degradation is due to the severe photocorrosion of BiVO_4_ (Fig. 3d, Supplementary Fig. 5a, c). In sharp contrast, the battery using α-Fe_2_O_3_ air photoelectrode achieves a stable cycling performance with the charge and discharge potential plateaus at ~1.64 and ~1.15 V, respectively, under illumination during the cycles for ~50 h (Fig. 5f). This corresponds to an impressively high energy efficiency of ~70.3% compared to the α-Fe_2_O_3_-based zinc–air battery of ~54.5% in the dark condition.

To further analyze the limiting factor for the long-term cycling of α-Fe_2_O_3_-based zinc–air battery, the new reassembled zinc–air battery was fabricated based on the disassembled α-Fe_2_O_3_ electrode from the failed battery by replacing the fresh KOH electrolyte and new zinc electrode. Interestingly, the new reassembled zinc–air battery can further cycle for ~40 h (Supplementary Fig. 11), indicating the excellent stable performance of α-Fe_2_O_3_ electrode, which is not the limiting factor for the long-term cycling. According to the working mechanisms of the battery, reactions of Zn electrode during the discharge process of zinc–air battery are accompanied with consumption of OH^−^ and formation of insoluble ZnO, leading to passivation of Zn electrode (reactions (6)–(7)), which will increase the resistance of the zinc–air battery and make it failure to discharge. Therefore, the limiting factor for the long-term cycling of zinc–air battery is the passivation of zinc electrode in alkaline electrolyte. The structure and morphology of two samples after the cycling test are further explored. The transmission electron microscopy (TEM) images for α-Fe_2_O_3_ and BiVO_4_ materials before and after cycling tests are analyzed. It can be seen that the surface morphology of BiVO_4_ electrode exhibits significant dissolution after cycling test compared to the initial electrode, but the high-resolution TEM (HRTEM) images of the BiVO_4_ electrode remains a lattice spacing of 0.46 nm, which is well consistent with the (011) plane of crystalline BiVO_4_ (Supplementary Fig. 12). With respect to the α-Fe_2_O_3_ electrode, the TEM images for α-Fe_2_O_3_ before and after cycling test show the same nanorod morphology (Supplementary Fig. 13). Moreover, the HRTEM images (Supplementary Fig. 13) of α-Fe_2_O_3_ before and after cycling display lattice fringes with an inter-planar spacing of 0.25 nm, matching well with the (110) plane of α-Fe_2_O_3_. The TEM results for α-Fe_2_O_3_ and BiVO_4_ materials after long-term cycling tests further indicate the high stability of α-Fe_2_O_3_. In addition, XPS tests for photoelectrodes before and after cycling test are conducted to understand the possible chemical changes of the materials. The XPS for BiVO_4_ before and after the cycling performance is provided in Supplementary Fig. 1a–c, which shows the remarkable decrease of XPS peak intensity, suggesting the sever dissolution of BiVO_4_ electrode during the cycling test. In contrast, there is no noticeable change in XPS spectra of α-Fe_2_O_3_ after stability test (Supplementary Fig. 1d–f), indicating the well maintained chemical compositions. Moreover, based on the EIS results of photoelectrodes (Supplementary Fig. 14), the charge-transfer resistance of BiVO_4_ increases compared to the initial electrode (Supplementary Fig. 3) after cycling test especially under illumination, which is close to that in the dark. This is due to the severe photocorrosion in the BiVO_4_, resulting in the performance degradation. In contrast, the charge-transfer resistance of α-Fe_2_O_3_ electrode remains almost the same after cycling test compared to the initial electrode (Supplementary Fig. 3), indicating its remarkable stability. The above characterization results for α-Fe_2_O_3_ and BiVO_4_ materials after long-term cycling tests further highlight the performance stability of α-Fe_2_O_3_. Furthermore, after the full discharge of 66 h, the α-Fe_2_O_3_-based zinc–air battery could be charged back under illumination. The recharged battery can continue to function over further cycling tests under illumination although there is a small decay of charge and discharge voltage which is due to the long-term consumption of zinc and electrolyte (Supplementary Fig. 15), demonstrating the sunlight-promoted charge process. Furthermore, we have replaced zinc electrode with a copper current collector for further confirming the sunlight-promoted charging process. Interestingly, it can be seen that Zn is deposited onto the copper current collector for the α-Fe_2_O_3_-based zinc–air battery after sunlight-promoted charging of 3 h at 1 mA cm^–2^. According to the XRD results (Supplementary Fig. 16a), the deposited Zn is indexed to metallic Zn (JCPDS Card No. 04–0831). All the other peaks are indexed to be metallic Cu (JCPDS Card No. 04–0836) from the copper substrate. In addition, the composition of deposited Zn is analyzed by energy-dispersive X-ray spectroscopy (EDX) (Supplementary Figs. 16b, c), which also suggests the successful deposition of Zn on the copper current collector, again confirming the sunlight-promoted charging process. All the results clearly highlight the promising α-Fe_2_O_3_ photoelectrode as an efficient air electrode utilizing solar energy for sunlight-promoted zinc–air battery, and also demonstrate that the band structure and the photoelectrochemical/chemical stability of photoelectrode play a significant role in determining the battery performance including the charging performance, energy efficiency and lifetime. As discussed above, the concept of sunlight-promoted OER in rechargeable zinc–air battery offers an effective strategy by utilizing solar energy to facilitate the OER kinetics of metal–air battery toward its wide application in future. To demonstrate its promising application in other metal–air batteries, we assembled the Fe–air battery based on the photoelectrode (e.g., α-Fe_2_O_3_) as an example. The “light-response” charging feature of the Fe–air battery is shown in Supplementary Fig. 17. Excitingly, the sunlight-promoted rechargeable Fe–air batteries exhibit charge potential of ~1.26 V based on α-Fe_2_O_3_. Once the sunlight is cut off, it can be seen that the applied voltage increases immediately. The example of the sunlight-promoted Fe–air batteries further demonstrates that the concept of utilizing solar energy to improve the oxygen evolution reaction kinetics can be extended to other metal–air batteries for addressing the charging overpotential issue.

## Discussion

In conclusion, we have successfully incorporated solar energy capturing into the charging process of a rechargeable zinc–air battery which integrates the semiconducting photoelectrode as air electrode. Under sunlight illumination, the photoelectrode undergoes photoexcitation and generates holes and electrons. The holes transfer to the photoelectrode surface to facilitate OER process, thus reducing the charging voltage. The proposed sunlight-promoted charging mechanism has been demonstrated using two typical photoelectrodes (i.e., BiVO_4_ and α-Fe_2_O_3_) with different band structures. BiVO_4_-based zinc–air battery can be initially charged with an extremely low voltage of ~1.20 V corresponding to ~0.76 V reduction compared to that in dark. However, it increases quickly due to severe photocorrosion of BiVO_4_. In contrast, α-Fe_2_O_3_ exhibits promising potential as stable and efficient air photoelectrode with a low charge potential of ~1.43 V and high cycling stability during 50 h discharge–charge test. It is also interesting to note that the difference in the actual charge potential (~0.23 V) of sunlight-promoted zinc–air batteries based on two different photoelectrodes matches well with the predicted theoretical value difference (0.20 V). This work demonstrates that the introduction of photoelectrode with more negative CB and appropriate VB positions as well as high photoelectrochemical stability is promising for the development of zinc–air batteries with high energy efficiency.

## Methods

### Electrode preparation

The porous BiVO_4_ photoelectrode was synthesized through electrodeposition of BiOI film on fluorine-doped tin oxide (FTO) substrate followed by thermal conversion to the porous BiVO_4_ film^20^. Briefly, 0.4 M KI solution was prepared by dissolving 20 mmol potassium iodide (KI, ≥99%, Sigma-Aldrich) into 50 ml deionized water and adjust its pH to 1.7 by nitric acid (HNO_3_, 65−68%, Sinopharm Chemical Reagent Beijing Co., Ltd, China). Then, 2 mmol bismuth(Ш) nitrate pentahydrate (Bi(NO_3_)_3_·5H_2_O, ≥99.99%, Sigma-Aldrich) was added into the solution and stirred by 15 min. Afterwards, this solution was mixed with 20 ml of absolute ethanol containing 0.4 M p-benzoquinone (≥98%, Sigma-Aldrich), and was vigorously stirred for 30 min resulting in a dark transparent black solution with a pH of 2.6. A typical three-electrode system was carried out for the electrodeposition, with the prepared solution as electrolyte, a FTO substrate as the working electrode (WE), a platinum foil as the counter electrode (CE) and the saturated Ag/AgCl as the reference electrode. After potentiostatically cathodic deposition at an optimized condition of **–**0.1 V vs. Ag/AgCl for 5 min, a uniform BiOI film was deposited on the FTO substrate followed by rinsing with deionized water and drying in ambient air. BiVO_4_ electrodes were prepared by dropping 0.2 ml of a dimethyl sulfoxide (DMSO, ≥99.7%, Sigma-Aldrich) solution containing 0.2 M vanadyl acetylacetonate (VO(acac)_2_, 99.98%, Sigma-Aldrich) onto the formed BiOI layer, followed by annealing in a muffle furnace at 450 °C (ramping rate 2 °C min^**−**1^) for 2 h. To remove excess V_2_O_5_, the obtained BiVO_4_ electrodes were soaked in 1 M potassium hydroxide (KOH, ≥85%, Sinopharm Chemical Reagent Beijing Co., Ltd, China) after cooling to room temperature. Finally, the electrodes were rinsed with deionized water (18.2 MΩ cm, Millipore) and dried in ambient air to obtain pure BiVO_4_.

α-Fe_2_O_3_ photoelectrode with one dimensional nanorod arrays on the FTO substrate was prepared by a hydrothermal method and two-step annealing. Typically, a cleaned FTO glass was immersed in a Teflon-lined stainless steel autoclave containing aqueous solution of 0.15 M ferric chloride (FeCl_3_, 99.99%, Sigma-Aldrich) and 1 M sodium nitrate (NaNO_3_, ≥99%, Sigma-Aldrich) at pH 1.25 adjusted by hydrochloric acid (HCl, ≥37%, Sinopharm Chemical Reagent Beijing Co., Ltd, China). The hydrothermal reaction was conducted at 95 °C for 6 h and naturally cooled down to room temperature. The obtained products were cleaned with deionized water and subsequently sintered in air at 550 °C for 2 h to form Fe_2_O_3_ nanorod arrays. Afterwards, the formed Fe_2_O_3_ product was further annealed at 800 °C for 10 min for facilitating the doping of Sn from the FTO into Fe_2_O_3_ to increase the donor density in the electrode.

### Characterization

The crystal structures of the obtained samples were determined by XRD (Bruker D8, Bruker Corp, USA) with Cu K_α_ radiation and operating in a 2*θ* range of 20–80° at a scan rate of 5° min^**−**1^. The surface morphology of the samples was characterized by field-emission SEM (Hitachi S-4800, Japan) and TEM (JEOL 2100 F, Japan). The Optical absorption measurements of as-prepared samples were performed using a spectrophotometer (Hitachi U-3010, Hitachi, Japan). VB analysis was carried out by XPS (Kratos Axis Ultra DLD, Kratos Analytical Ltd., UK). UPS was measured with a monochromatic He I light source (21.2 eV) and a VG Scienta R4000 analyzer (VG Scienta Ltd., UK). A sample bias of −5 V was applied to observe the secondary electron cutoff. The work function can be determined by the secondary electron cutoff at the high kinetic energy region.

### Photoelectrochemical measurements

The photoelectrochemical and electrochemical performances of the photoelectrode were measured with potentiostat (Ivium Stat, Ivium Technologies, Netherlands) in a three*-*electrode configuration using BiVO_4_ or α-Fe_2_O_3_ as the working electrode, saturated calomel electrode (SCE) as reference electrode, graphite rod as counter electrode and 1 M KOH with pH of 13.6 as electrolyte. In order to ensure the dark environment, the quartz electrolytic cell is wrapped in tin foil. Simulated solar illumination was provided by a 500 W Xe lamp equipped with an AM 1.5 G filter (CEL-S500, Beijing China Education Au-light Co. Ltd., China) and the incident photo intensity was calibrated to 100 mW cm^**−**2^ by a solar power meter. Liner sweep voltammetry (LSV) was carried out in O_2_-saturated electrolyte with scanning rate of 20 mV s^**−**1^ in dark and under simulated solar illumination, respectively. Potentials were calibrated to a RHE using the Nernst equation:

13 $$\begin{array}{l}E\left( {{\mathrm{versus}}\,{\mathrm{RHE}}} \right) = E\left( {{\mathrm{versus}}\,{\mathrm{SCE}}} \right) + E_{{\mathrm{SCE}}}\left( {{\mathrm{reference}}} \right) + 0.0591\,{\mathrm{V}} \times {\mathrm{pH}}\\ \left( {E_{{\mathrm{SCE}}}\left( {{\mathrm{reference}}} \right) = 0.244\,{\mathrm{V}}\,{\mathrm{versus}}\,{\mathrm{NHE}}\,{\mathrm{at}}\,25\,^\circ \!{\mathrm{C}}} \right)\end{array}$$

The photocurrent stability tests were conducted under continuous illumination at 1.23 V vs. RHE. Electrochemical impedance spectroscopy (EIS) measurements were performed in a three*-*electrode configuration using the electrochemical station with bias of 1.23 V vs. RHE in the dark and under illumination (AM 1.5 G; 100 mW cm^**−**2^), respectively. Mott–Schottky measurements were carried out in 1 M KOH aqueous solution at a frequency of 1 kHz with a scan rate of 10 mV s^**−**1^ in the dark. The donor densities were calculated with the following equation:

14 $$N_{\mathrm{D}} = \left( {2/e\varepsilon \varepsilon _o} \right)\left[ {{\mathrm{d}}\left( {1/C^2} \right)/{\mathrm{d}}V} \right]^{ - 1}$$

where *N*_d_ is the donor concentration, *e* the electron charge (1.60 × 10^**−**19^), *C* the electron charge, *ε* the dielectric constant (*ε* *=* 68 for BiVO_4_^38^, and *ε* *=* 80 for α-Fe_2_O_3_^39^, *ε*_*o*_ the vacuum permittivity (8.85 × 10^**−**14^ F cm^**−**1^), *C* the capacitance of the space charge region, and *V* is the electrode applied potential.

### Sunlight-promoted zinc–air batteries assembly

The zinc–air battery tests were performed by the home-made liquid zinc–air battery which was assembled using a polished Zn foil (0.25 mm thickness) as the metal electrode and the semiconductor photoelectrode (i.e., BiVO_4_ or α-Fe_2_O_3_) as the air electrode. The electrolyte used was 1 M KOH with 0.03 M zinc acetate (Zn(CH_3_COO)_2_, 99.99%, Sigma-Aldrich) (dissolved in KOH to form zincate, ${\mathrm{Zn}}\left( {{\mathrm{OH}}} \right)_4^{2{\mathrm{ - }}}$) to ensure reversible Zn electrochemical reactions at the Zn electrode. For comparison, the performance of sunlight-promoted rechargeable zinc–air batteries was tested under the same conditions as traditional zinc–air batteries except the illumination on the photoelectrode. The galvanostatic discharge–charge cycling tests (20 min discharge followed by 20 min charge) were performed by Land-CT2001A battery-testing system (Wuhan LAND Electronic Co., Ltd, China) in the dark and under illumination, respectively. The specific capacity (mAh g^**−**1^) and the energy density (mWh g^**−**1^) based on the weight of consumed Zn are calculated according to Eqs. 15 and 16:

15 $${\mathrm{Specific}}\,{\mathrm{capacity}} = \frac{{{\mathrm{Discharge}}\,{\mathrm{current}} \,\times {\mathrm{Service}}\,{\mathrm{hours}}}}{{{\mathrm{the}}\,{\mathrm{weight}}\,{\mathrm{of}}\,{\mathrm{consumed}}\,{\mathrm{Zn}}}}$$

16 $${\mathrm{Energy}}\,{\mathrm{density}} = \frac{{{\mathrm{Discharge}}\,{\mathrm{current}} \,\times {\mathrm{Service}}\,{\mathrm{hours}} \times {\mathrm{Average}}\,{\mathrm{discharge}}\,{\mathrm{voltage}}}}{{{\mathrm{the}}\,{\mathrm{weight}}\,{\mathrm{of}}\,{\mathrm{consumed}}\,{\mathrm{Zn}}}}$$

## Supplementary information

Supplementary Information

## Data Availability

The data that support the findings of this study are available from the corresponding author upon reasonable request.
