# Supplementary material for: Utilizing solar energy to improve the oxygen evolution reaction kinetics in zinc–air battery
Source: Nat Commun. 2019 Oct 18;10:4767. doi: 10.1038/s41467-019-12627-2 (PMC6800449; doi:10.1038/s41467-019-12627-2)
Supplement: Supplementary file 1 — Supplementary Information [file 41467_2019_12627_MOESM1_ESM.pdf]

Supplementary Information for

Utilizing solar energy to improve the oxygen evolution reaction kinetics in zinc–air battery

Liu et al.

Supplementary Figures

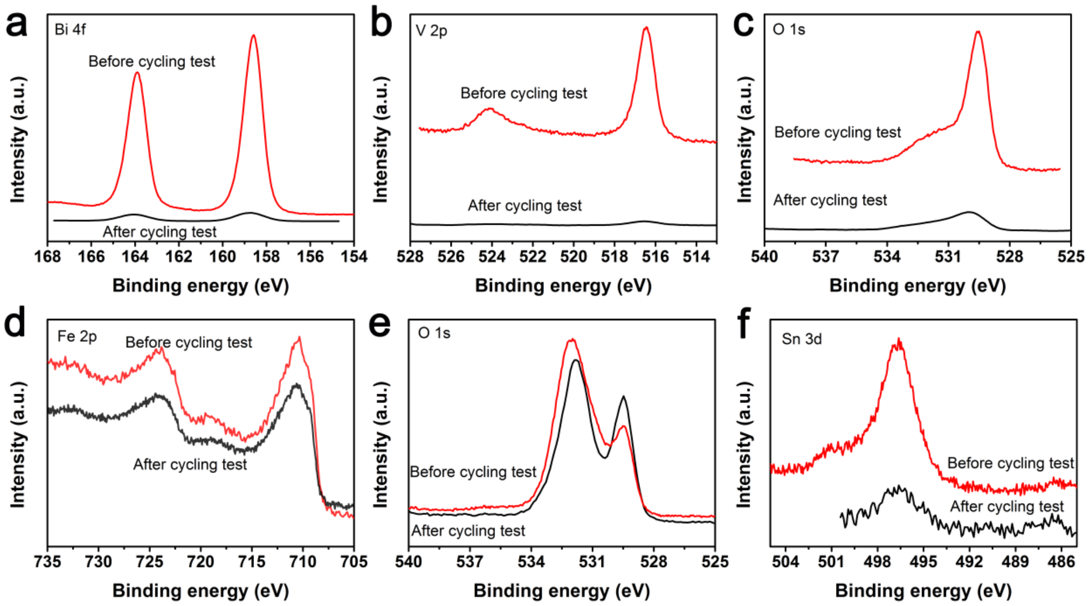

Supplementary Figure 1. XPS spectra of $BiVO_4$ and α-Fe_2O_3 photoelectrodes before and after cycling test. High resolution XPS spectra of **a** Bi 4f and **b** V 2p **c** O 1s from $BiVO_4$ photoelectrode before and after cycling test. **d** Fe 2p and **e** O 1**s** **f** Sn 3d from the α-Fe_2O_3 photoelectrode.

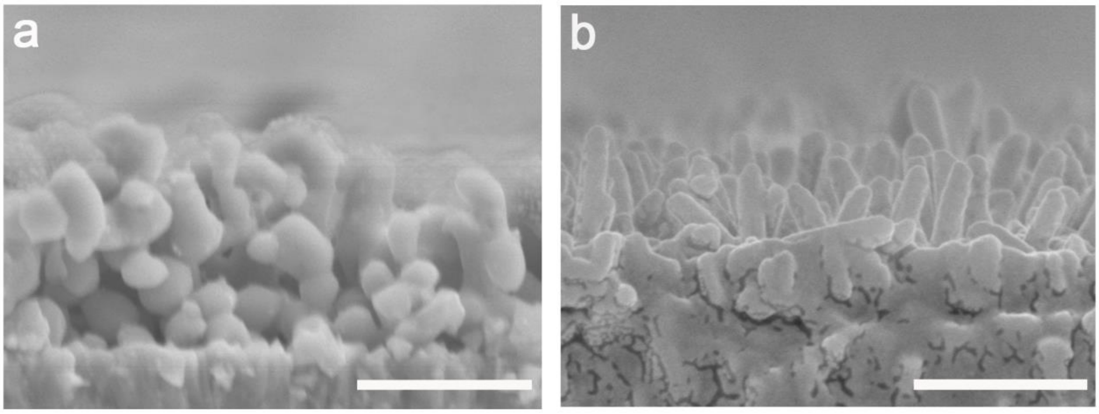

Supplementary Figure 2. Cross-sectional images of photoelectrodes. Cross-sectional images of **a** α-Fe_2O_3 and **b** $BiVO_4$. Scale bars in **a**, **b** are 500 nm.

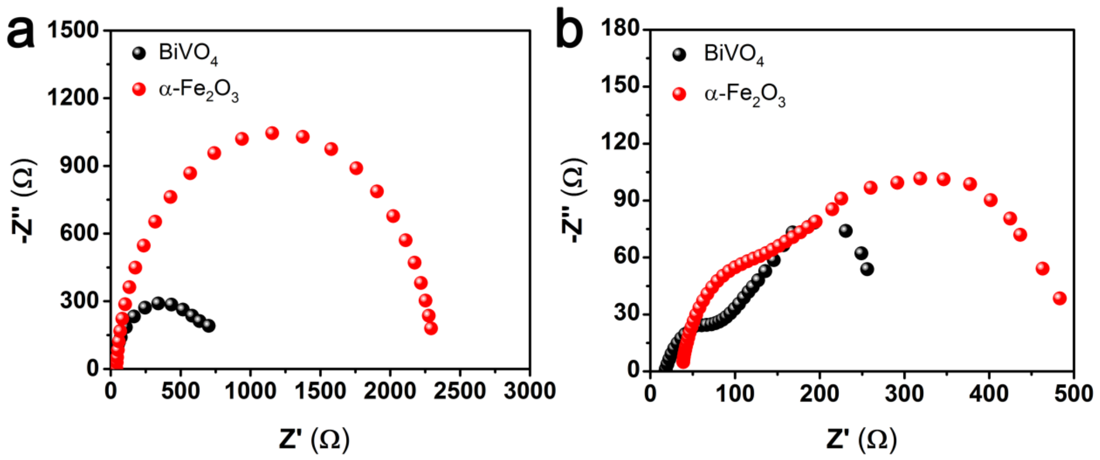

Supplementary Figure 3. Nyquist plots of photoelectrodes. Nyquist plots of $BiVO_4$ and α-Fe_2O_3 photoelectrodes attained at 1.23 V (versus RHE) **a** in the dark and **b** under illumination.

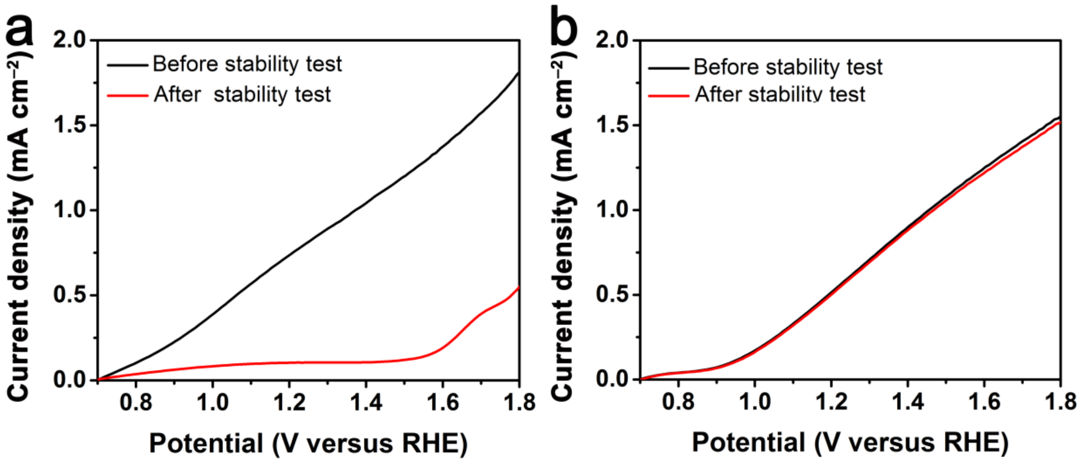

Supplementary Figure 4. Photoelectrochemical performance of the photoelectrodes. Current density–potential curves of **a** $BiVO_4$ and **b** α-Fe_2O_3 before and after current density–time stability tests under illumination.

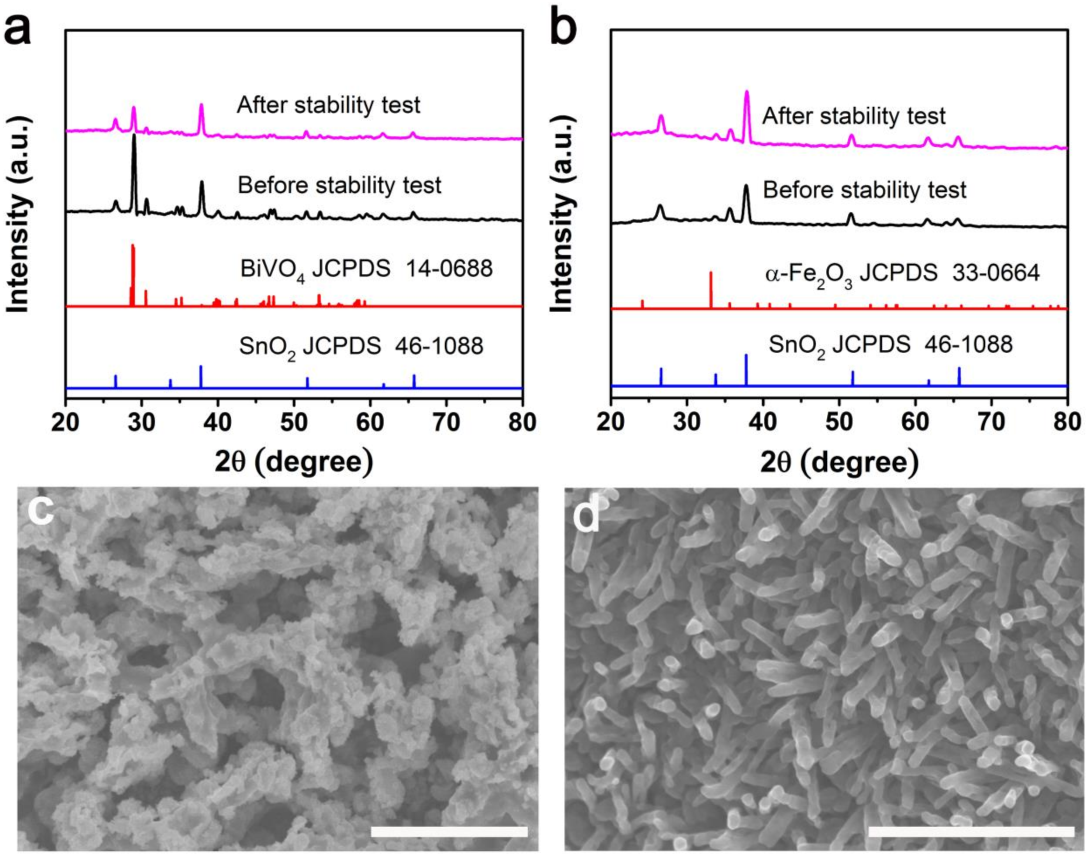

Supplementary Figure 5. Characterization of the photoelectrodes. XRD patterns of **a** $BiVO_4$ and **b** α-Fe_2O_3 before and after stability test. SEM images of **c** $BiVO_4$ and **d** α-Fe_2O_3 after stability test. Scale bars in **c**, **d** are 1 μm.

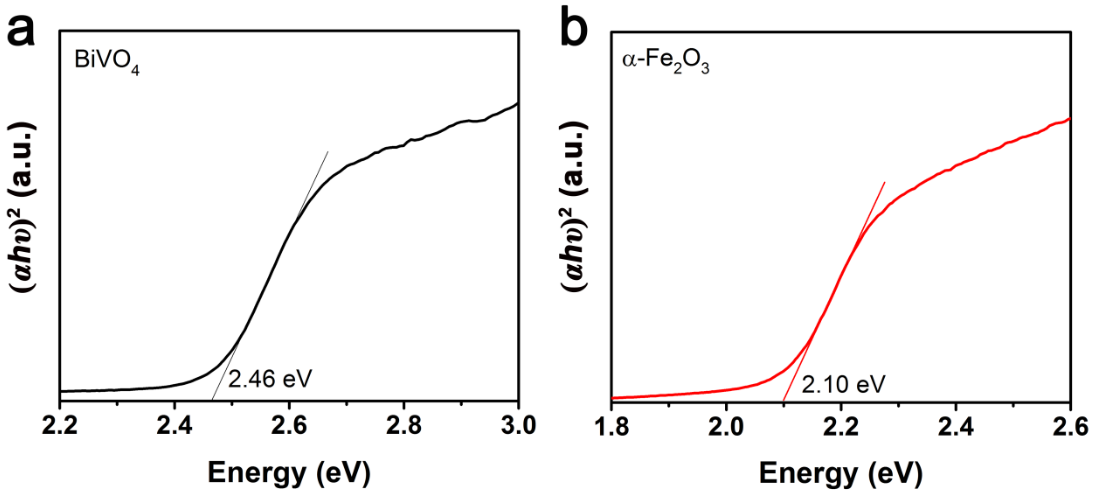

Supplementary Figure 6. Band structures of the photoelectrodes. Estimated band-gap energy of **a** $BiVO_4$ and **b** α-Fe_2O_3.

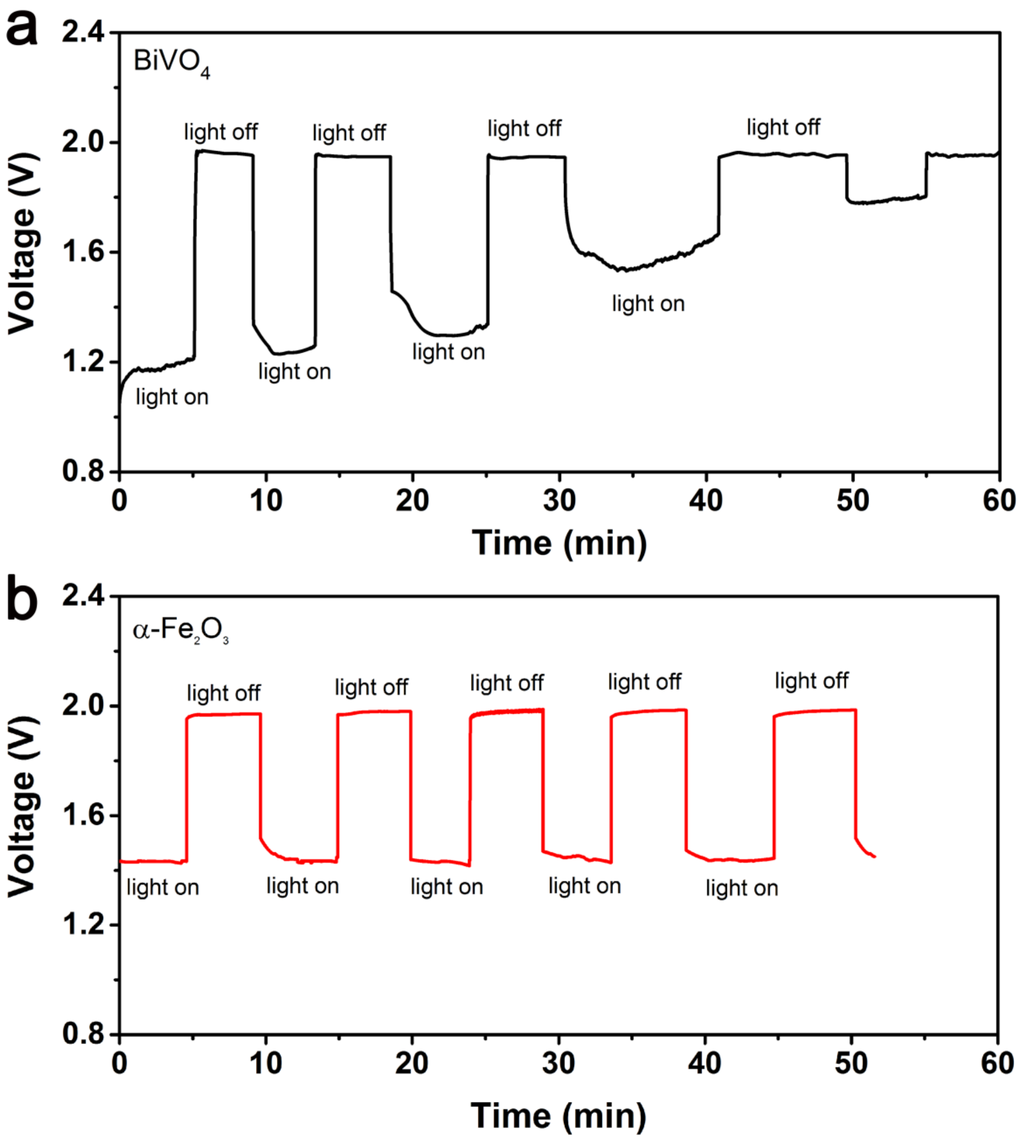

Supplementary Figure 7. Light responses of the sunlight-promoted rechargeable zinc–air batteries with photoelectrodes. Light responses of the sunlight-promoted rechargeable zinc–air batteries with **a** $BiVO_4$ and **b** α-Fe_2O_3 as the photoelectrode at the current density of 0.1 mA cm^{-2}.

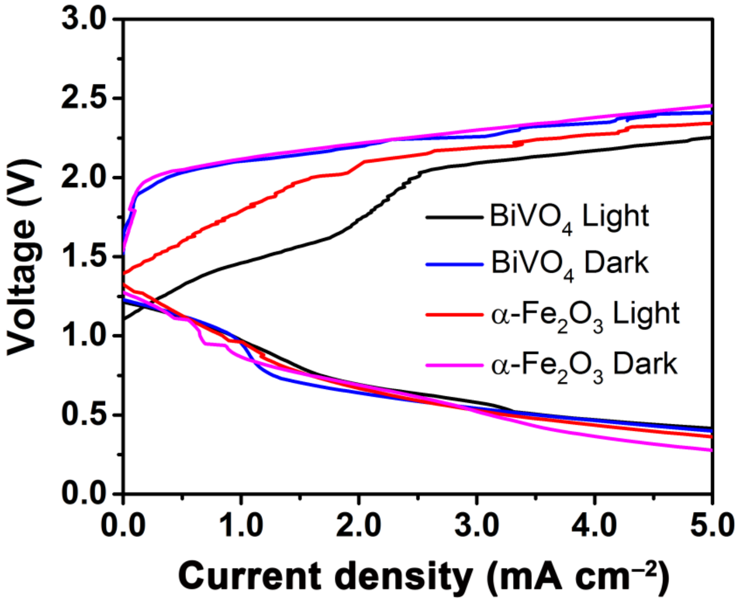

Supplementary Figure 8. The discharge–charge polarization curves for the sunlight-promoted rechargeable zinc–air battery based on $BiVO_4$ and α-Fe_2O_3 in the dark and under illumination.

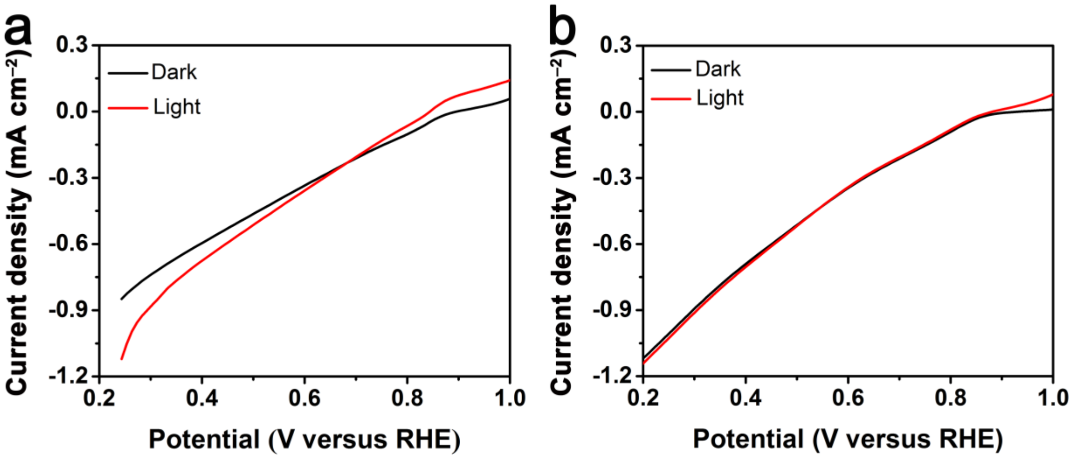

Supplementary Figure 9. ORR polarization plots. ORR polarization plots of **a** $BiVO_4$ and **b** α-Fe_2O_3 measured in the dark and under illumination.

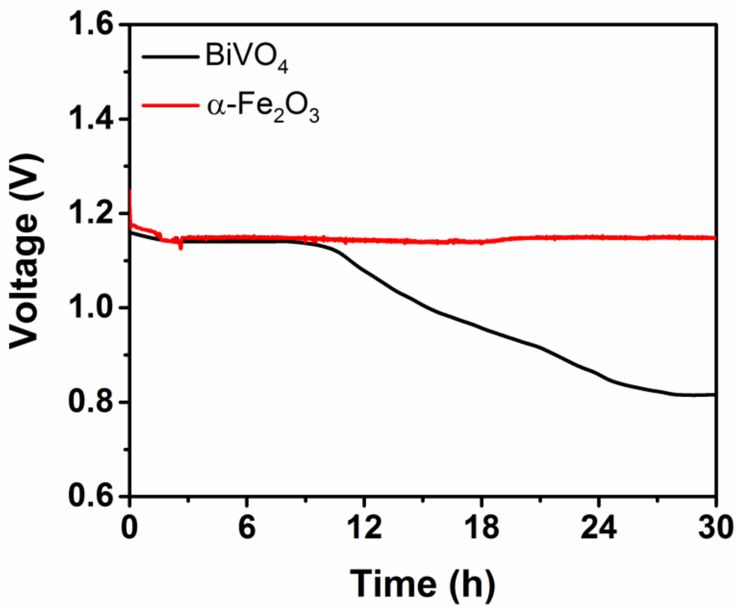

Supplementary Figure 10. The galvanostatic discharging curves for the sunlight-promoted rechargeable zinc–air battery at the current density of 0.5 mA cm^{-2}.

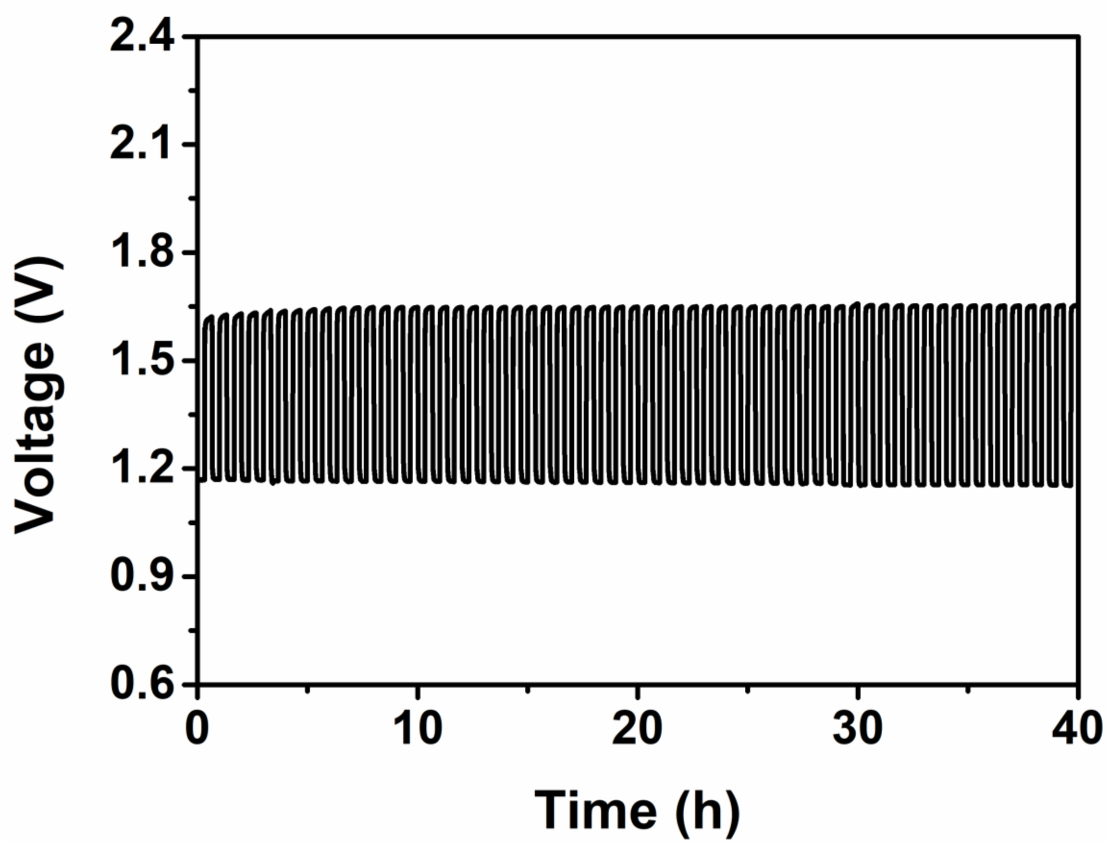

Supplementary Figure 11. Cycling performance of sunlight-promoted rechargeable zinc–air battery under illumination with dissembled α-Fe_2O_3 air photoelectrode by replacing the fresh KOH electrolyte and new zinc electrode, at a current density of 0.5 mA cm^{-2}.

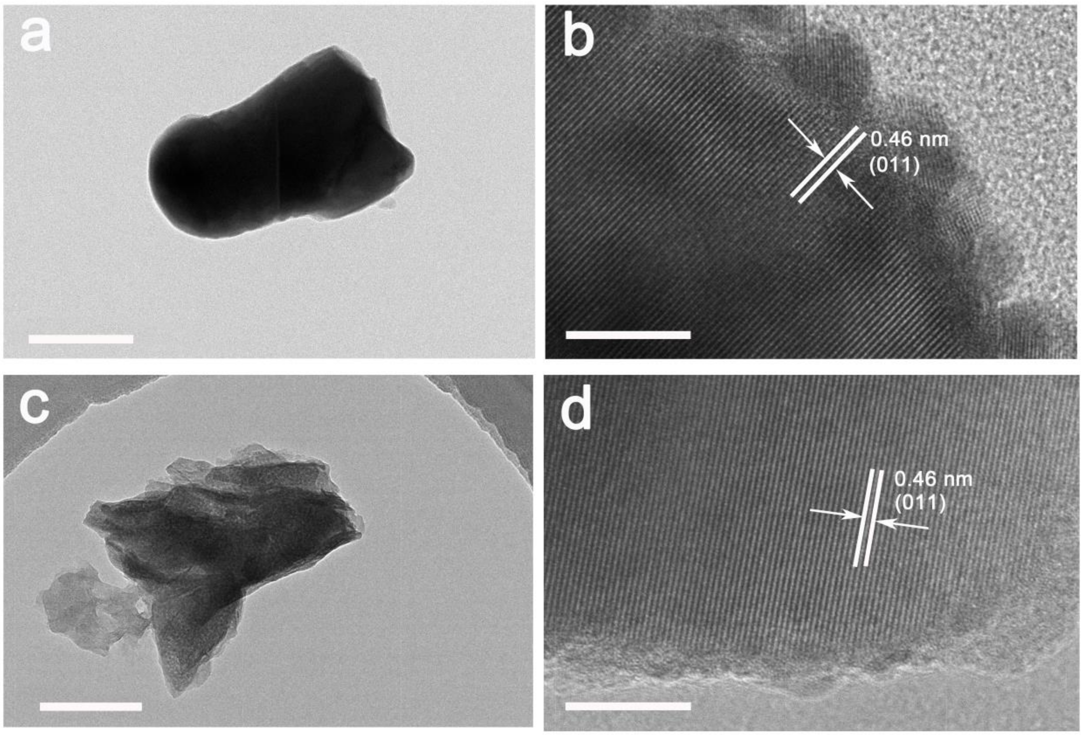

Supplementary Figure 12. TEM images of $BiVO_4$ air photoelectrode before and after cycling test. TEM images of $BiVO_4$ air photoelectrode **a** before and **c** after cycling test. HRTEM images of $BiVO_4$ air photoelectrode **b** before and **d** after cycling test. Scale bars in **a**, **c** are 100 nm and in **b**, **d** are 10 nm.

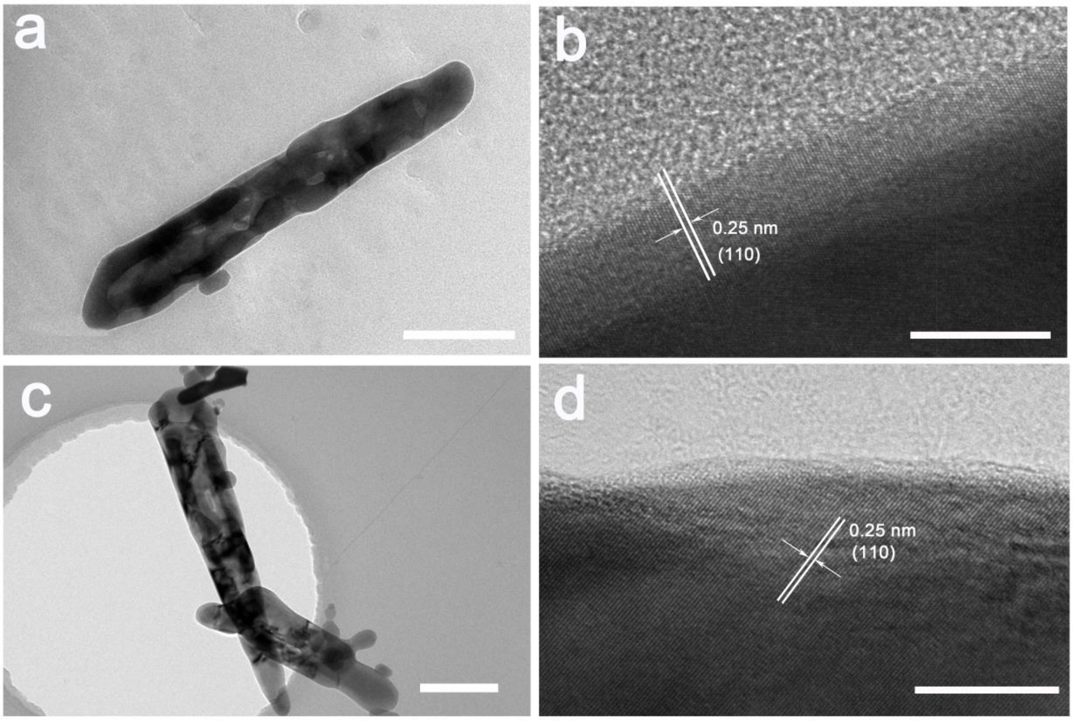

Supplementary Figure 13. TEM images of α-Fe_2O_3 air photoelectrode before and after cycling test. TEM images of α-Fe_2O_3 air photoelectrode **a** before and **c** after cycling test. HRTEM images of α-Fe_2O_3 air photoelectrode **b** before and **d** after cycling test. Scale bars in **a**, **c** are 100 nm and in **b**, **d** are 10 nm.

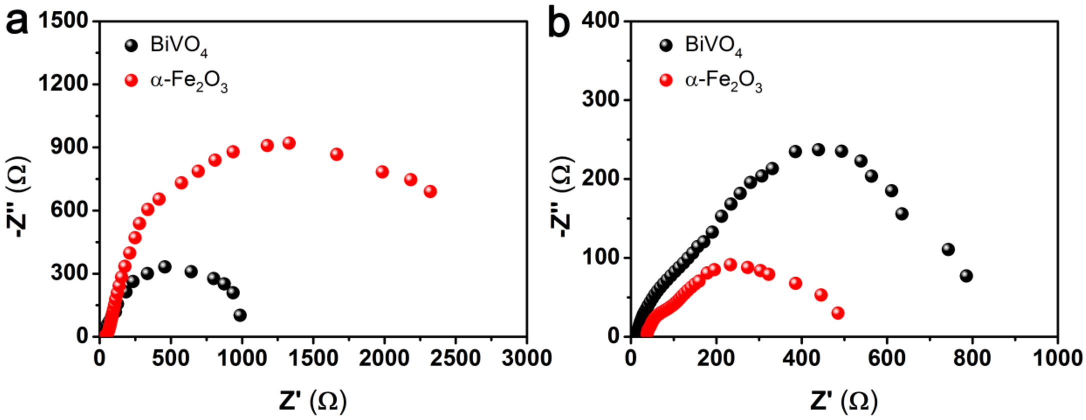

Supplementary Figure 14. Nyquist plots of photoelectrodes after cycling test. Nyquist plots of $BiVO_4$ and α-Fe_2O_3 photoelectrodes attained at 1.23 V (versus RHE) **a** in the dark and **b** under illumination after cycling test.

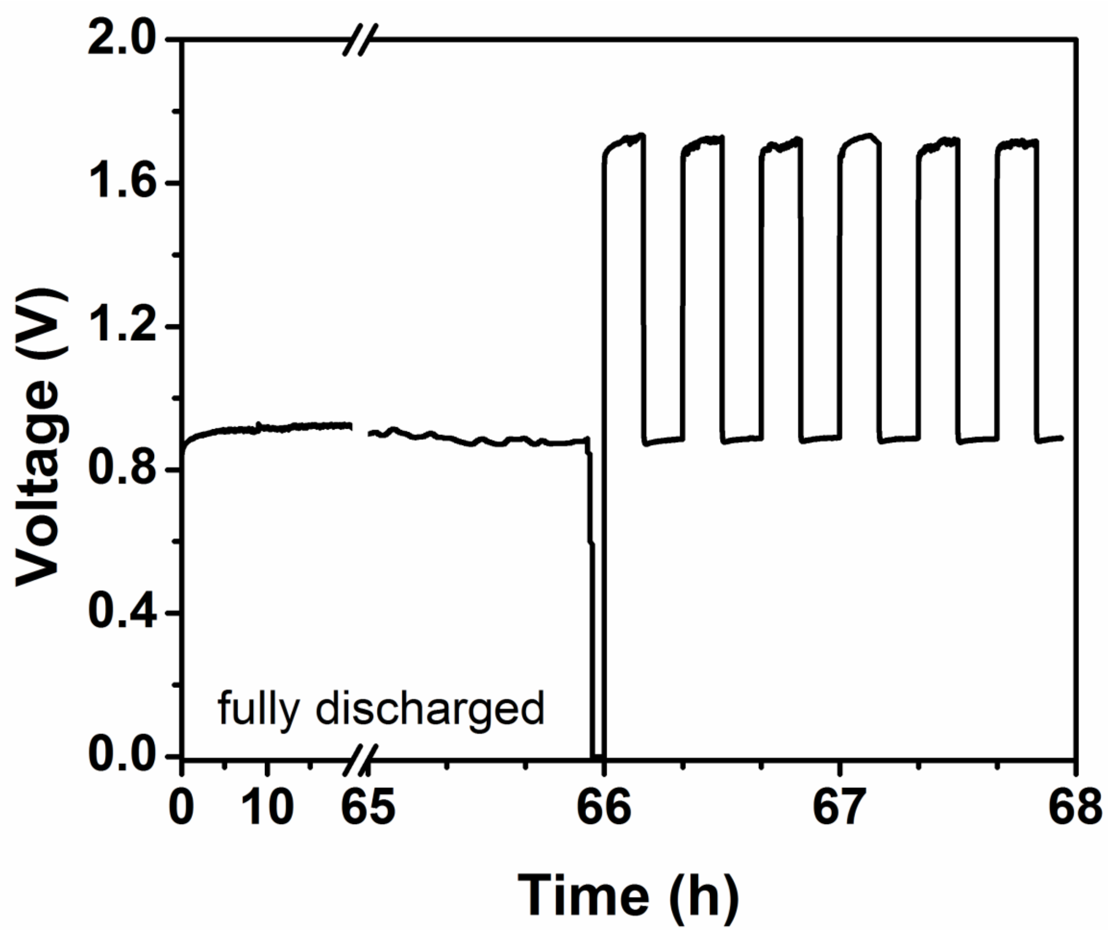

Supplementary Figure 15. The cycling performance of the sunlight-promoted rechargeable zinc–air battery based on α-Fe_2O_3 air photoelectrode at the current density of 0.5 mA cm^{-2} under illumination after fully discharged at a current density of 1 mA cm^{-2}.

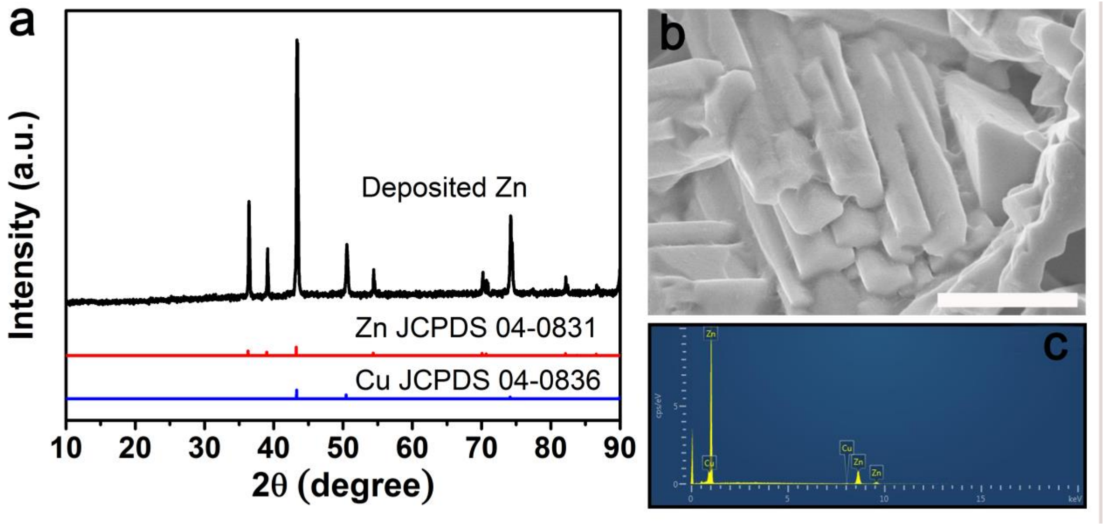

Supplementary Figure 16. Characterizations of deposited Zn on the copper collector after sunlight-promoted charging. a XRD patterns and **b** SEM image, **c** EDX analysis of deposited Zn on the copper collector after sunlight-promoted charging of 3 h at 1 mA cm^{-2} for the zinc–air battery based on the α-Fe_2O_3 photoelectrode. Scale bars in **b** are 2 μm.

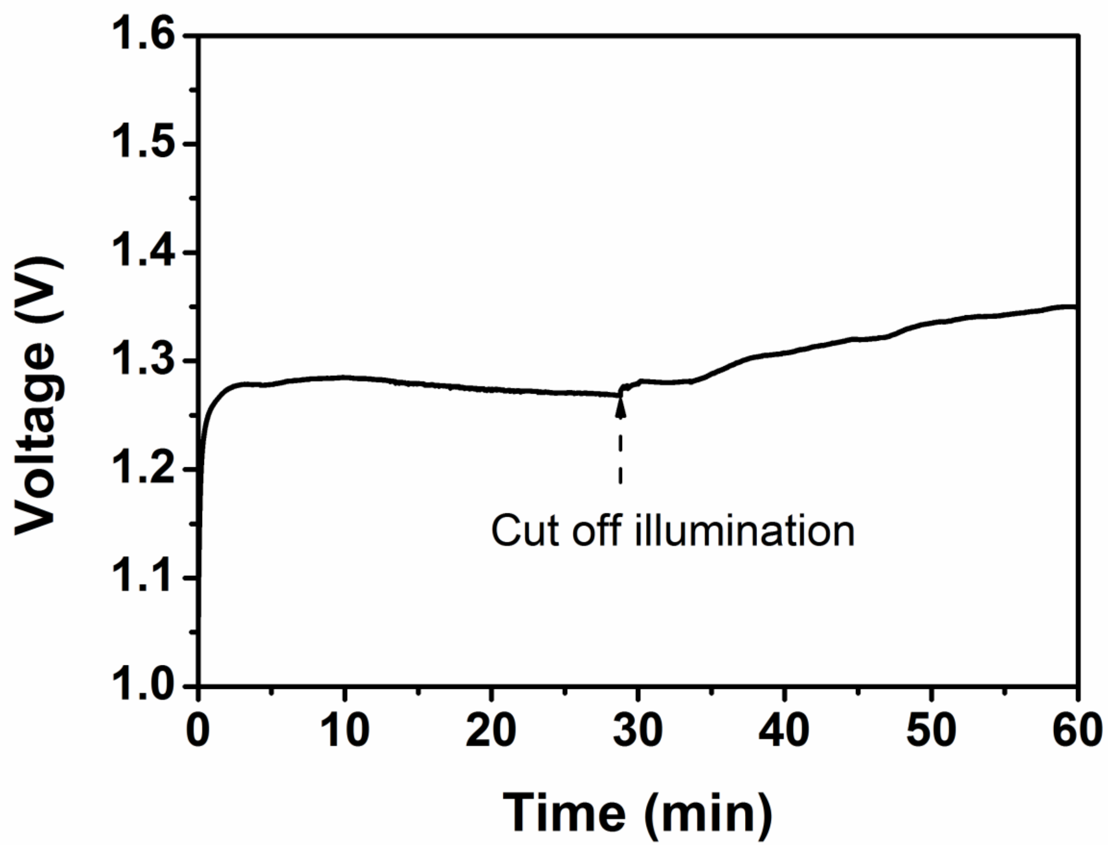

Supplementary Figure 17. Light response of the charging process for Fe–air battery.

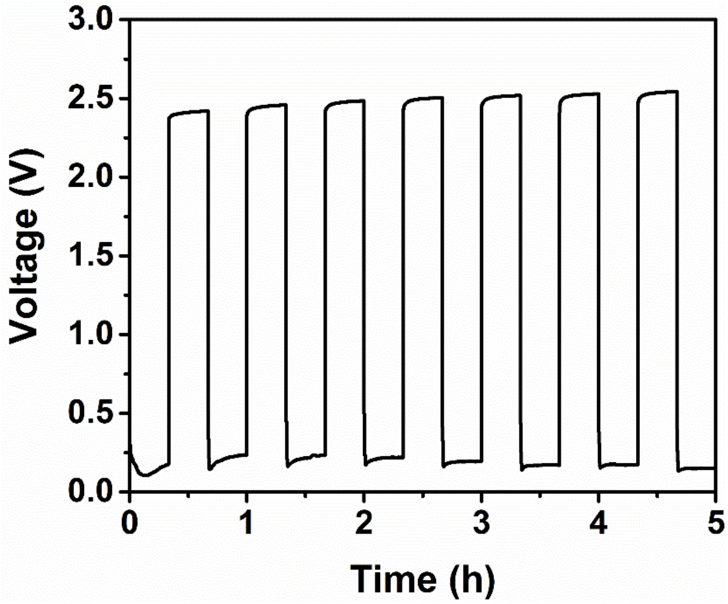

Supplementary Figure 18. The discharge–charge curves for the rechargeable zinc–air battery with bare FTO electrode as the air electrode at the current density of 0.5 mA cm^{-2} under simulated AM 1.5G illumination.

Supplementary Table 1. Comparison of charge voltages of sunlight-promoted zinc–air batteries based on different photoelectrodes in the dark and light and the calculation of energy savings.

Photoelectrodes	Charge voltage in dark	Charge voltage in light	Energy savings
$BiVO_4$	1.96 V	1.20 V	38.8%
α-Fe_2O_3	1.97 V	1.43 V	27.4%

Supplementary Notes

As shown in supplementary Fig. 18, in the absence of catalysts, the rechargeable zinc–air battery with bare FTO air electrode shows a charge potential of ~2.5 V under illumination, which is much higher than that of sunlight-promoted rechargeable zinc–air battery based on the air photoelectrodes (i.e., $BiVO_4$ and α-Fe_2O_3). Simultaneously the discharge potential is very low of less than 0.3 V. It can be concluded that bare FTO has little effect on the sunlight-promoted charging process in the rechargeable zinc–air battery.
